# Adhesive Gelatin-Based Eutectogels: A Review of Synthesis, Properties, and Applications

**DOI:** 10.3390/polym18020222

**Published:** 2026-01-14

**Authors:** Raluca Ioana Baron, Andreea Laura Chibac-Scutaru, Gabriela Biliuta, Sergiu Coseri

**Affiliations:** “Petru Poni” Institute of Macromolecular Chemistry of Romanian Academy, 41A, Grigore Ghica Voda Alley, 700487 Iasi, Romania; andreea.chibac@icmpp.ro (A.L.C.-S.); biliuta.gabriela@icmpp.ro (G.B.)

**Keywords:** gelatin, deep eutectic solvent, eutectogel, adhesive properties

## Abstract

This review presents a focused assessment of the rapidly expanding field of gelatin-based eutectogels and identifies the gaps in current literature that justify this examination. Research on deep eutectic solvents (DESs and NADES) has advanced quickly, yet there is still no integrated view of how these solvent systems influence adhesion in gelatin-based gels. Eutectogels are soft materials formed by gelling DESs or NADES with biopolymers. Gelatin is widely used because it is biocompatible, biodegradable, and readily available. We provide a clear overview of the chemistry of DESs and NADES and describe how gelatin forms networks in these media. The review summarizes established knowledge on adhesion, highlighting the contributions of polymer network density, interfacial hydrogen bonding, and solvent mobility. New perspectives are introduced on how these factors interact to control adhesion strength, toughness, and reversibility. A key topic is the role of hydrogen bond donors (HBDs) and acceptors (HBAs). They define the hydrogen bonding environment of the solvent and represent an underexplored way to tune mechanical and adhesive behavior. Examples such as moisture-resistant adhesion and temperature-responsive bonding show why these systems offer unique and adjustable properties. The review concludes by outlining major challenges, including the lack of standardized adhesion tests and constraints in scalable production, and identifying directions for future work.

## 1. Introduction

Flexible electronics are cutting-edge electronic parts made on pliable substrates that can change shape without losing their functionality. Advanced materials such as conductive polymers, organic semiconductors, and nanomaterials enable this versatility, producing robust and lightweight devices that can be used in a wide range of applications. Their significance is especially noticeable in bionic robotics, wearable medical devices, smart packaging, and human–machine interfaces [1,2,3,4]. Flexible electronics provide real-time vital sign tracking and continuous health monitoring through comfortable gadgets in wearable healthcare systems, encouraging proactive health management and improved adherence among patients. By incorporating flexible sensors that provide tactile feedback, these electronics in bionic robots, allow for a more human-like interaction with their surroundings, improving assistive and rehabilitation technology. Despite significant advancements in soft sensors and actuators, it remains exceptionally difficult to create intelligent soft materials that can both actuate and sense their own motions, mimicking the neuromuscular behaviors of living organisms. Strain sensors made with stretchable polymeric matrices and conductive fillers have been used intensively. These sensors are employed due to their high stretchability, direct current (DC) compatibility, ease of fabrication, and ability to be tuned to have a specific resistance that is dependent on the degree of connection to the conductive filler network. Carbon black [5], carbon nanotubes [6], graphene derivatives [7], and liquid metals [8], have been reported as efficient conductive fillers capable of providing high sensitivity and an extended detection range, reaching strains of up to 400% [9,10]. In recent years, other materials with remarkable properties have been investigated, such as two-dimensional MXene-type carbides [11], flexible metal particles (e.g., Ag, Cu) dispersed in elastomeric matrices [12], ionic conductive hydrogels [13], and self-healing polymers with tuned conductivity [14,15]. However, during fabrication, the immiscible filler particles in the composite are naturally attracted to each other, resulting in the formation of aggregates. This hinders the adaptive movements and causes irreversible damage to the device [16,17]. To overcome these shortcomings, researchers have sought alternative solutions that meet the industry’s ever-growing needs. One of the most effective solutions for achieving consistent, reliable and robust electrical conductivity over time and with repeated use is the use of deep eutectic solvents (DESs). These solvents represent an emerging class of ionic media, obtained by combining a hydrogen bond donor (HBD) and a hydrogen bond acceptor (HBA), which form a low-melting eutectic mixture and a complex network of intermolecular interactions [18]. DESs are distinguished by properties such as high thermal stability, non-volatility, significant ionic conductivity and environmental compatibility. In addition, they can act simultaneously as plasticizers and conductive media, promoting uniform dispersion of filler particles and reducing the formation of aggregates. This versatility makes them promising candidates for integration into soft composites, strain sensors and flexible bioelectric devices [19,20,21]. Conversely, the polymers that comprise such assemblies are pivotal to ensuring functionality. There are growing efforts to use natural polymers from the polysaccharide and protein classes. These polymers have key advantages, including biodegradability, bioavailability, cost, renewability, and structural variability [22]. Thus, polysaccharides such as cellulose and its derivatives, often those resulted by a selective oxidation reaction [23], as well as chitosan [24], starch [25], alginate [26] and pullulan [27], are widely used. Due to their dense hydrogen bond networks, these materials exhibit excellent filmogenicity, controlled permeability, and ability to interact with ionic species in DES. Furthermore, their chemical structure can be easily modified by esterification, etherification, or functionalization with ionic groups, which allows for fine-tuning of electrochemical properties [28]. Because they are renewable, they may be produced continuously using a wealth of natural resources. With an annual production of about 160 billion tons, plant biomass has great promise for obtaining polysaccharides from cellulosic materials [29]. Since polysaccharides are biodegradable, they break down without harming the environment, meeting eco-friendly standards and lessening their influence on the environment. Gelatin is a product derived from collagen, a protein that is found in animals. Gelatin is unique in that it is the only non-carbohydrate thickening agent used in food. Gelatin is non-toxic and biodegradable. This protein is inexpensive and has the ability to produce hydrogels [30]. In addition, gelatin–polysaccharide-based products have demonstrated a wide range of advantageous properties in both the food and non-food sectors, such as biodegradable packaging, engineering and bio functional sensors. Due to their complementary nature, these hybrid systems combine the flexibility and film-forming properties of gelatin with the chemical stability and three-dimensional network formation capacity of polysaccharides. Films, hydrogels, foams and micro- and nanoscale particles have been successfully produced from such gelatin–polysaccharide blends, demonstrating excellent performances in terms of elasticity, water retention, selective permeability and biological compatibility [31,32]. The interactions that occur between proteins and polysaccharides are complex and determine the final properties of the material. These include hydrogen bonds, steric exclusion forces, hydrophobic and electrostatic interactions, which can vary significantly depending on pH, ionic strength and mixing ratio [33,34]. Of these, electrostatic interactions play a particularly important role in the formation of soluble or insoluble complexes between positively charged proteins (such as gelatin below its isoelectric point) and anionic polysaccharides (e.g., alginate, pectin, carboxymethylcellulose). These complexes can be controlled by adjusting the pH, which allows for the achievement of various structures—from homogeneous solutions to stable gelled networks. Furthermore, products based on gelatin—polysaccharides have demonstrated numerous advantageous properties in both the food and non-food sectors. Films, hydrogels, and nano- and microparticles have all been successfully produced using gelatin—polysaccharide systems. Among the many interactions that occur between proteins and polysaccharides are hydrogen bonds, steric exclusion, and hydrophobic and electrostatic interactions. Strong electrostatic complexes are common and pH-dependent in protein-polysaccharide matrices. Complexation, cosolubility, and segregation are the three distinct matrices that can be created when protein and polysaccharide are mixed [35]. More recently, these gelatin—polysaccharide matrices have been integrated with DES and conductive agents (e.g., metal ions, organic salts, carbon nanoparticles), leading to the formation of ionic hydrogels and soft conductive composites capable of combining biomimetic mechanical properties with stable electrical functionalities [21,36]. These hybrid materials offer promising prospects for advanced applications in the fields of wearable electronics, strain sensors, bioelectrodes, and controlled drug delivery systems [37].

This review holds particular significance as it coherently synthesizes contemporary knowledge regarding the application of DES in conjunction with natural polymers, with a specific focus on gelatin and polysaccharide-based systems. Although each of these components has been extensively studied individually, a systematic analysis correlating the interaction mechanisms, structural properties and functional performances of these materials has been lacking until now [20]. In a global context where sustainability, biodegradability and energy efficiency are becoming strategic priorities, these hybrid systems offer a promising alternative to conventional synthetic materials, opening new research directions in the field of soft electronics and green materials. This review aims not only to synthesize existing knowledge, but also to identify emerging trends, unsolved challenges, and development opportunities in the design of bioinspired materials with advanced electrical functionalities. Thus, the paper provides a useful reference framework for both researchers exploring fundamental phenomena at the DES—biopolymer interface and those developing practical applications in areas such as flexible sensors, biomedical platforms, and sustainable wearable devices.

Although significant progress has been achieved in exploring both DES and natural polymers, the current state of research remains fragmented, often lacking a unified understanding of how these systems interact at different structural levels. Many studies have focused on material synthesis and characterization without fully elucidating the mechanisms that govern their functional behavior in complex environments [20]. This critical gap limits the rational design and large-scale application of sustainable soft materials. Consequently, this review seeks to bridge these inconsistencies by providing an integrative and analytical perspective that connects fundamental interactions with practical performance. Beyond consolidating existing findings, it aims to outline new conceptual directions and experimental strategies capable of advancing DES–biopolymer systems toward intelligent, eco-efficient, and multifunctional platforms suitable for next-generation flexible electronics.

## 2. Deep Eutectic Solvents (DESs)

**Figure 1:**
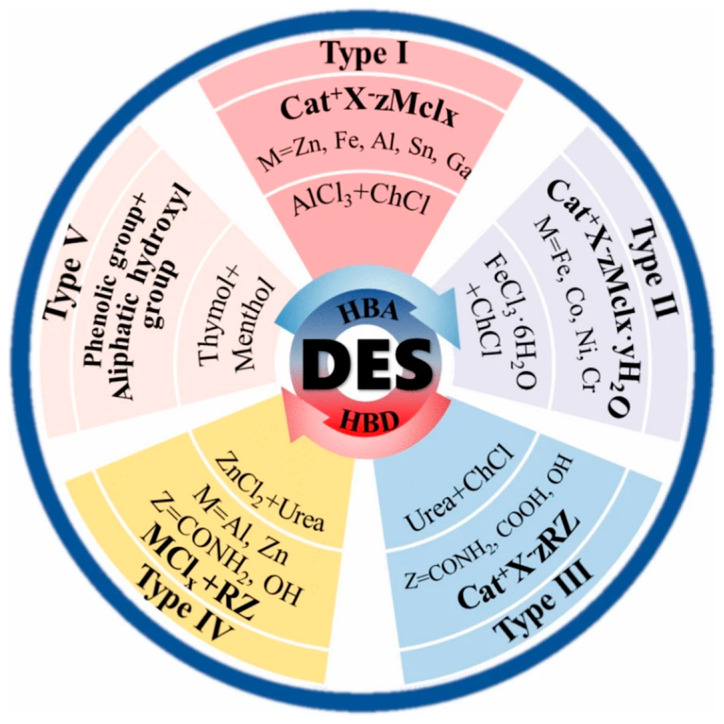
General classification of DESs. The figure is reproduced from [40].

DESs have emerged as a sustainable alternative to ionic liquids in biomass pre-treatment, overcoming critical limitations such as high cost and poor biodegradability [38]. In 2003, Abbott et al. first reported the choline chloride-urea low-temperature eutectic system [18], demonstrating its unique ability to dissolve metal oxides and formally establishing the concept of “deep eutectic solvents”. This pioneering work signaled the start of comprehensive DESs studies. By 2006, Abbott et al. [39] expanded the use of the choline chloride-urea DES, facilitating the dissolution of cellulose and ushering in a new era of DESs utilization in many fields [40]. DESs are simpler to prepare and less costly than ionic liquids. They are often made from biodegradable or naturally occurring substances. These characteristics, combined with their tunable physicochemical properties, make DESs attractive for a wide range of applications, including green chemistry, materials science, biomedicine, and pharmaceuticals [41]. DESs are typically synthesized in the following structural configurations: Cat^+^X^−^*z*Y, X stands for a Lewis base, usually a halide anion, Y for a Lewis or Brønsted acid, and z for the number of molecules interacting with the anion. The cation (Cat) is commonly an amine, phosphonium, or sulfonium. The classification of DESs is summarized in a large portion of the literature [42], and it may be subdivided into five groups according to its constituent parts (Figure 1). DESs have definite environmental advantages over ionic liquids (ILs), especially when it comes to lifecycle sustainability and biodegradability. DESs are usually made of natural, low-toxicity ingredients like choline chloride, organic acids, or sugars, in contrast to conventional ILs, which are primarily made from petrochemical feedstock and are frequently linked to high toxicity to organisms and poor biodegradability [43]. Because of this design basis, DESs are naturally less hazardous to the environment. Standardized OECD testing classifies DESs as “readily biodegradable,” which stands in stark contrast to ILs ecological concessions [44].

In general terms, DESs are considered to be specific mixtures of HBDs and HBAs that exhibit lower melting points compared to their individual components, as a result of the increase in system entropy, mainly determined by the complex network of hydrogen bonds between HBDs and HBAs [45]. DESs have several advantages, including simplified preparation methods, negligible vapor pressure, non-flammability, biocompatibility, and biodegradability [46]. The specific benefits of a given DES depend on its formulation. The robust hydrogen bonding interactions between HBDs and HBAs lend these solvents considerable potential for use with various solutes and in different chemical processes. The most widely used HBA is choline chloride. It is an inexpensive, biodegradable compound (over 93% within 14 days) with very low acute toxicity [47]. It is used, for example, as a dietary component for animals (vitamin B4). The DES systems summarized in Table 1 represent the most frequently cited combinations in DES research and provide a useful basis for understanding solvent–biopolymer interactions relevant to this study. Their ability to disrupt the hierarchical structure of lignocellulosic biomass—through solvation of lignin, cleavage of hemicellulose linkages, and swelling of crystalline cellulose—has made them increasingly attractive for sustainable polymer and biomass processing. The chemical diversity of DES allows fine-tuning of solvent performance through simple variation of the HBA:HBD ratio, enabling their adaptation to specific extraction, dissolution, or pretreatment tasks.

DESs are exceptional alternatives to ILs in polysaccharide dissolution, providing additional advantages such as cost-effectiveness, simple preparation, and non-toxicity [54]. Despite the numerous investigations conducted on the dissolution of polysaccharides in DESs, researchers frequently encounter difficulties in achieving high solubility and sustainable synthetic protocols in producing DES-containing polysaccharide-based eutectogels [55].

## 3. Adhesion as an Essential Quality of Gels: General Principles and Examples

Adhesion is one of the fundamental properties of gels, defining their ability to establish and maintain stable contact with another surface through physical or chemical interactions [56]. In soft materials and biofunctional systems, adhesive behavior plays an essential role in determining the overall performance, being directly correlated with the stability, flexibility and functionality of gel-based devices [56,57]. Thus, adhesion is a critical parameter for applications such as bioadhesives, smart dressings, deformation sensors, wearable patches or controlled drug delivery platforms [56,58]. The adhesive properties of gels derive from a complex balance between the chemical composition of the polymers, the three-dimensional network architecture, the degree of hydration, and the nature of the contact surface [56]. The predominant interactions include hydrogen bonds, van der Waals forces, electrostatic interactions, and, in some cases, dynamic covalent bonds [59]. Natural polymers, such as gelatin, chitosan, alginate, or pectin, are particularly effective in developing adhesive properties due to the presence of reactive functional groups—hydroxyl, carboxyl, and amino—that can form reversible bonds with biological or synthetic surfaces [58]. In particular, gelatin has emerged as a reference material in the study of adhesive behavior, due to its ability to form flexible gel networks and establish multiple interactions at the interface [60]. Through the presence of amino and carboxyl groups, gelatin can generate strong interactions with biological tissues, which makes it a frequently used component in surgical bioadhesives, implantable hydrogels and gelatin–polysaccharide composites with controlled adhesion [60]. In addition, the adhesive properties of gelatin can be adjusted by chemical modification (e.g., gelatin methacrylate—GelMA) or by combining with DES, which improve flexibility, stability and interfacial compatibility [61]. The adhesion of gels also depends on external conditions, such as pH, temperature, humidity, and applied compression force, which can influence the mobility of polymer chains and the energy of interactions at the interface [59]. Depending on these parameters, gels can exhibit reversible adhesive behavior—useful in temporary applications, such as skin patches—or irreversible, characteristic of permanent fixation systems [56]. Next, the general principles governing adhesion mechanisms in gels are discussed, along with representative examples of gelatin–polysaccharide systems and other bioinspired materials, in which this property is a determining factor for the final performance of the material.

### 3.1. Adhesive Strengths

One of the mandatory requirements for eutectogels with applicability in bioelectronics is their ability to adhere to skin or other substrates. Researchers have particularly focused their recent studies on investigating this property. Eutectogels based on gelatin and various eutectic solvents exhibited a remarkable combination of adhesion, conductivity, and mechanical flexibility. Table 2 presents examples of these systems.

A systematic comparison of recent studies highlights clear correlations between formulation strategy and material performance. Systems incorporating double-network architectures or polymeric reinforcers such as PVA or TOCNF report the highest mechanical strength, reaching tensile values up to 6.8 MPa in Chen et al. [62] and T-peel strengths above 5 MPa in Zhang et al. [66]. In contrast, supramolecular eutectogels with very low gelatin content, such as those described by Qiao et al. [63], maintain good flexibility but show moderate strength. Adhesion displays strong dependence on interfacial hydrogen bonding and the presence of polyphenolic crosslinkers: TA-reinforced eutectogels (Mercadal et al. [67]) exhibit higher adhesion energy than CNC-only systems, whereas ternary-DES formulations (Xu et al. [36]) provide broad substrate compatibility, including PTFE, due to abundant polar and ionic interactions. Conductivity varies significantly depending on whether the system supports pure ionic transport (DES-based) or mixed ionic-electronic conduction, as reported by Picchio et al. [64] for PEDOT-containing eutectogels. The variability among studies can be traced to differences in DES composition (ratio of HBD/HBA), network density, type of conductive additives, and testing conditions. These comparisons illustrate how formulation choices govern mechanical stability, wet adhesion, and electrical performance, highlighting both strengths and limitations across the current literature.

**Figure 2:**
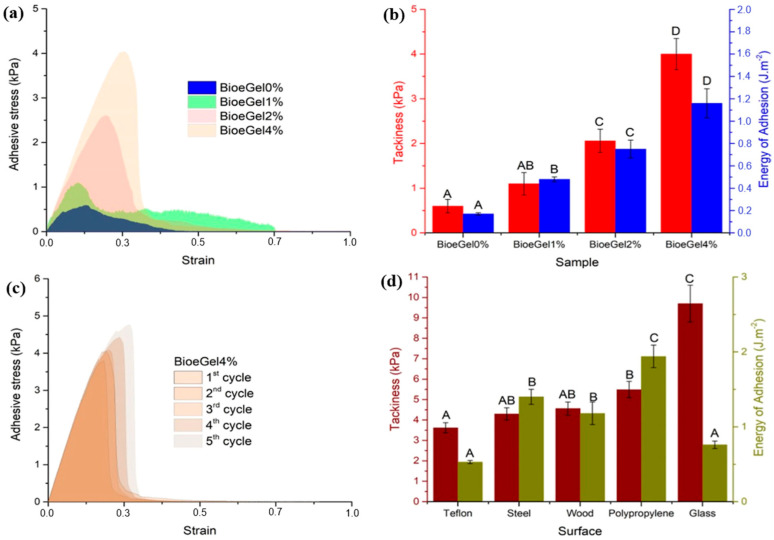
(**a**) Adhesive stress vs. strain curves of the BioeGels. (**b**) Tackiness (red column) and adhesion energy (blue column) values of BioeGels. (**c**) Cyclic adhesive stress vs. strain curves of the prepared BioeGel4%. (**d**) Tackiness (brown column) and adhesion energy (gold column) values in different surfaces of BioeGel4%. Two bar values with the same letter are not significantly different (*p* ≥ 0.05) according to Tukey’s test. The figure is reproduced from [67].

More recent research has investigated formulations that combine gelatin and DES, and sometimes conductive polymers or double networks, with a particular focus on evaluating adhesion in real-world environments (different materials, varying temperatures, high humidity, etc.). Thus, Mercadal explored the ability of self-adhesion on the skin of tannic acid-decorated cellulose nanocrystals (TA@CNC) as dynamic filler nanomaterials for gelatin-based eutectogels composed of choline chloride and ethylene glycol eutectic mixtures. The eutectogels demonstrated remarkable self-adhesion, a key requirement in the manufacture of conformational skin sensors. The determining component of the eutectogel’s adhesion property was TA@CNC, and increasing the amount of TA@CNC led to higher adhesion energy values due to a greater number of hydrogen bonds between gelatin, TA and CNC, as shown in Figure 2 [67].

The adhesion values reported in this study are lower than those for gelatin-based eutectogels reported by Mercadal et al. but higher than for CNC hydrogels, demonstrating that the adhesion is sufficient for application to the skin and suitable for multiple successive cycles [68,69]. A significant advantage of these eutectogels is their versatile adhesion, as they adhere to other surfaces as well (teflon, steel, wood, polypropylene, and glass), with values comparable to those obtained on skin, making them suitable for a wide range of applications in sensors and wearable or surface-mounted devices.

**Figure 3:**
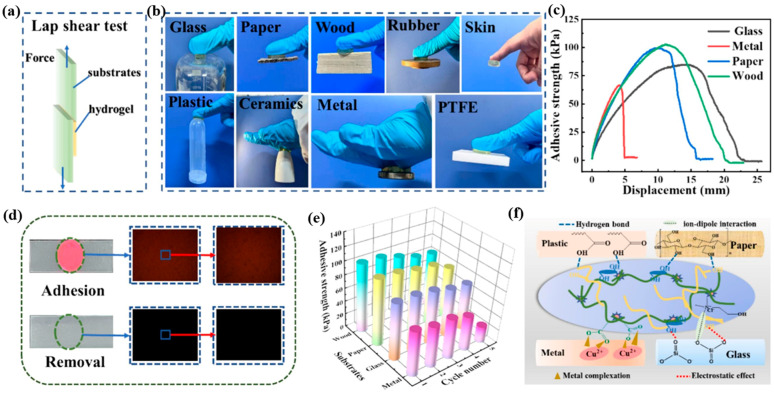
Adhesion properties of DESG eutectogels. (**a**) Schematic diagram of DESG eutectogels adhesion test; (**b**) Photographs of DESG eutectogels adhered to different substrates; (**c**) Fluorescence microscope image of DESG eutectogels adhesion-peeling off a silicone rubber plate after staining with rhodamine B; (**d**) Overlap-shear adhesion curves of DESG eutectogels adhesion to different substrates; (**e**) Repeatable adhesion strength curves of DESG eutectogels adhesion to different substrates; (**f**) Possible interfacial interactions between DESG eutectogels and different substrates. The figure is reproduced from [36].

Similar results were reported by Lina Xu et al., who obtained multifunctional ionically conductive eutectogels with double network structure based on gelatin and a ternary eutectic solvent composed of acrylic acid (AA), choline chloride (ChCl), and ethylene glycol (EG) [36]. These eutectogels, in addition to their remarkable mechanical properties, also exhibited excellent adhesion to a wide range of substrates (glass, wood, paper, rubber, leather, plastics, ceramics, metals, and polytetrafluoroethylene (PTFE)), Figure 3. The strong adhesion of the eutectogels came from the abundance of -OH groups in the DES, which are linked to the gelatin and PAA polymer chains and are greatly amplified by multiple physical interactions such as hydrogen bonds, ion–dipole interactions, metal complexes, and van der Waals interactions.

### 3.2. Mechanisms Controlling Gelatin-Based Gels’ Adhesion Features

Analyzing the literature, it can be concluded that the adhesion process of gelatin-based eutectogels to various substrates is governed by a synergistic combination of physicochemical mechanisms. These determine both the initial formation of the interfacial contact and the stability and reversibility of the bonds during mechanical stresses.

(i) Hydrogen bond networks—The amide, carboxyl and hydroxyl groups of gelatin interact with polar species of DESs, generating a dense network of reversible hydrogen bonds. These interactions contribute to the formation of stable contact with substrates such as glass, metals, polymers or skin, ensuring good surface agreement [70].
(ii) Ionic interactions and salt bridges—The ionic components of DES, especially ChCl and monomeric acids (such as acrylic acid), participate in the formation of electrostatic bridges between the gelatin chains and the adherent substrate. These Columbic interactions enhance the energy dissipation at the interface and increase the shear and exfoliation resistance [36].
(iii) Dynamic networks—The presence of peptide motifs or polyphenols (e.g., tannic acid) in the eutectogel matrix allows the formation of dynamic bonds that can break and re-form under mechanical stress. These bonds contribute significantly to the hardness and mechanical resilience of the material, allowing for efficient dissipation of interfacial energy and persistent adhesion over time [71].
(iv) Eutectic solvent effects—DES acts not only as an ionic medium but also as an adhesion-stabilizing agent. Due to its high hygroscopicity, DES suppresses gel drying and freezing, maintaining a conformal and stable contact at different temperatures and humidity levels—thus preventing one of the major causes of failure of conventional hydrogels [71].

Through the combined action of these mechanisms, gelatin-based eutectogels can achieve strong and reversible adhesion to a variety of substrates, making them promising materials for wearable sensors, bioelectronics devices, and temporary fixation systems.

A number of structural and compositional factors also influences the adhesion of these gels:

- Gelatin concentration—determines the network density and the number of active functional groups (–OH, –COOH, –NH_2_) [71];
- DES solvent composition—type of hydrogen bond donor/acceptor (choline, urea, organic acids) and their molar ratio influence cohesion energy and interfacial stability [36];
- Crosslinking agents—polyphenols (e.g., tannic acid) or peptide agents form additional networks that improve mechanical integrity and adhesion [67];
- Nanoscale fillers—cellulose nanocrystals (CNC) and nanofibers (CNF) increase the roughness of the interface and favor ionic transport, which strengthens the bonds at the interface [72].

By optimizing these factors, stable, elastic and remarkably adhesive eutectogels can be obtained, capable of maintaining high performance even under variable environmental conditions.

**Figure 4:**
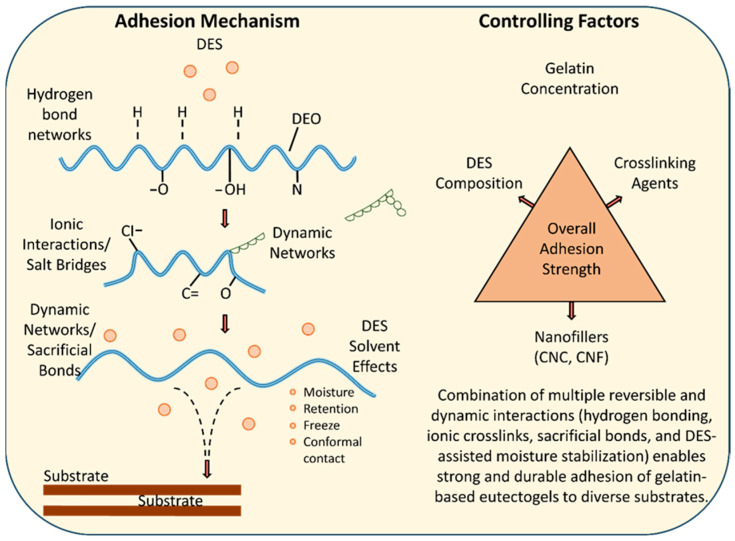
Schematic representation of adhesion mechanisms and controlling factors for gelatin-based eutectogels.

A schematic representation of the adhesion mechanisms and controlling factors for gelatin-based eutectogels is presented in Figure 4. The combination of reversible and dynamic interactions—hydrogen bonds, ionic bridges, sacrificial bonds and the stabilizing effects of DES—allows for strong and durable adhesion of gelatin-based eutectogels to a variety of substrates. The main factors influencing this property are the gelatin concentration, the DES composition, the nature of the crosslinking agents and the presence of CNC/CNF nanofibers.

### 3.3. Underwater Adhesives

**Figure 5:**
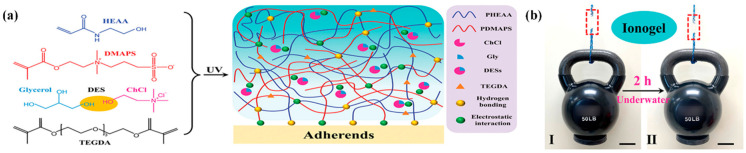
(**a**) Chemical structure and schematic diagram of the adhesive ionogel. (**b**) Adhesion strength of the ionogel on a polymethylmethacrylate substrate in water. The figure is reproduced from [77].

Submersible adhesives that can bond to wet surfaces or surfaces that are immersed in aqueous media are in great demand due to their wide range of possible applications in biomedical adhesion, the marine sector, underwater repair, sensors, and water-based adhesives, underwater repair, submersible sensors, and water-based energy equipment. These adhesives are indispensable for the development of flexible, tough, and durable materials that can function in extreme humidity conditions without loss of mechanical performance or interface stability [73]. However, adhesion in wet environments remains a fundamental challenge, as the presence of water layers at the interface prevents direct contact between the adhesive and the substrate, reducing interaction forces and mechanical cohesion [74]. To overcome this barrier, researchers have explored advanced ionogels systems based on DES, which can offer a combination of tunable hydrophilic and hydrophobic properties, as well as a complex network of positive and negative charges capable of establishing strong electrostatic interactions at the interface. This strategy is particularly effective because many solid surfaces in aqueous environments, such as glass, rocks, or metals, are negatively charged, which favors the formation of ionic bonds between DES cations and the substrate [75,76]. A notable example is the report of the group led by Khademhosseini, who developed an innovative ionogel by photopolymerization of zwitterions and polar monomers in a DES environment, completely replacing water or conventional organic solvents, Figure 5 [77]. The interfacial infiltration and adhesion strength were ensured by multiple hydrogen bonds and electrostatic interactions formed between the DES and the zwitterionic monomers. The cohesion strength was promoted by ultrafast gelation. The adhesion strength of the ionogel reached 1.04 MPa, which could lift a 50-lb dumbbell. Notably, the adhesion strength was maintained for more than 2 h after immersion in water (Figure 5b).

ChCl and glycerol, which acted as hydrogen bond donor and acceptor, respectively, were combined to form the translucent and viscous DES in this system. The ionic properties of the DES were enhanced by the electrostatic interactions of the N^+^ − Cl^−^ bonds. To create the first crosslinked network, polarized poly(*N*-hydroxyethyl acrylamide) (PHEAA) was chosen due to its exceptional endurance against corrosion and water. Since the co-presence of anions and cations made it easier to build a hydration shell without compromising hydrogen bonding, zwitterionic poly(*N*-(3-sulfopropyl)-*N*-(methacryloxyethyl)-N,N-dimethylammonium betaine) (PDMAPS) was incorporated to increase the ionogel’s underwater resilience [78]. Furthermore, the adhesion property under water was improved through multiple electrostatic interactions between the DES and PDMAPS [79]. PHEAA’s hydroxy and amino groups, glycerol’s hydroxy groups, and ChCl’s hydroxy groups all interacted with one another. Through hierarchical hydrogen bonding, this interaction improved the ionogel’s mechanical properties. With the quaternary ammonium cations (N^+^) of PDMAPS and ChCl, respectively, oxygen anions (O^−^) and chloride anions (Cl^−^) established electrostatic connections.

The combination of gelatin, DES and zwitterionic components offers a new paradigm for the development of green, biocompatible and high-performance adhesives, capable of stable operation in aquatic environments. By fine control of the chemical composition and interfacial interactions, these materials can be used to make flexible bioelectrodes, wearable sensors, adhesive medical patches and biodegradable surgical fixation devices. In the future, this research paves the way for a new generation of smart and renewable adhesives, capable of combining ecological sustainability with functional performance under extreme conditions [80].

## 4. DES-Gelatin Based Flexible Eutectogels

In recent years, the development of flexible and multifunctional gels has become a priority research direction in the field of soft materials, with applications ranging from wearable sensors and bioelectrodes to energy devices and smart biomedical platforms. Among all the emerging material categories, eutectogels based on DES and natural biopolymers such as gelatin have attracted particular interest due to their unique combination of sustainability, flexibility, conductivity and biocompatibility [18,81]. Eutectogels are materials in which a DES acts simultaneously as a solubilizing medium, plasticizer, and active ionic phase, while gelatin forms the three-dimensional network that confers cohesion and elasticity. Due to its structure rich in functional groups (–OH, –COOH, –NH_2_), gelatin can interact intensively with DES components through hydrogen bonds and electrostatic interactions, generating stable, homogeneous, and reversible structures. Such eutectogels exhibit superior mechanical properties and stability under variable environmental conditions, including low temperatures, where conventional hydrogels tend to freeze or shrink.

Compared to classical water-based hydrogels, gelatin–DES systems offer multiple advantages:

- Anti-freeze and anti-evaporation stability, due to the non-volatile and hygroscopic nature of DES;
- High ionic conductivity, useful in sensor and electroactive material applications;
- Self-healing and tuned adhesion, due to dynamic hydrogen bond networks;
- Biocompatibility and biodegradability, which make them suitable for medical and bioelectrical applications [8,36].

Gelatin-based eutectogels with DESs obtain their functional properties from the synergistic interactions between the ionic solvent phase and the gelatin polymer network. Unlike conventional water-based hydrogels—where water dictates transport and phase behavior—DESs create dense, hydrogen-bonded networks that strongly interact with both gelatin chains and residual water molecules. Antifreeze properties are due to strong hydrogen bonding between DES components and water, which disrupts the regular ordering of water into ice. Thermodynamic studies have shown that these donor–acceptor interactions significantly lower the chemical potential of water and prevent crystallization, resulting in freezing point depression even below zero degrees Celsius [82]. In gelatin-based eutectogels, this effect is further enhanced by the confinement of water within the polymer network, which further inhibits ice nucleation. The anti-evaporation stability of these gels results from the very low vapor pressure of DESs compared to water, as well as their high hygroscopicity [48]. Multiple hydrogen bonds form between DES components and water molecules, which reduces water diffusion and evaporation relative to standard aqueous hydrogels. Consequently, gelatin–DES eutectogels maintain their dimensional integrity and mechanical stability for long periods under ambient conditions. High ionic conductivity is achieved due to the inherently ionic nature of DESs, where charge carriers primarily migrate via a hydrogen-bond-assisted hopping mechanism, rather than the vehicular transport typical in water-based electrolytes [83]. The gelatin network acts as a mechanically stable yet permeable matrix, preserving continuous ion conduction pathways while limiting solvent loss. Self-healing and tunable adhesion arise from dynamic, reversible non-covalent interactions, such as multiple hydrogen bonds, ionic interactions, and, in some systems, metal–ligand coordination. When the material is damaged, these interactions can temporarily break and then reform, allowing the network to rearrange and recover its mechanical properties [84]. At interfaces, the same mechanisms enable strong adhesion to polar substrates and, in some cases, enhanced resistance to moisture.

To enable a broader comparison with prior studies, Table 3 summarizes representative gelatin–DES based eutectogels together with related DES-based and supramolecular hydrogel systems, highlighting general structure–property relationships relevant to anti-freezing behavior, ionic conductivity, and self-healing performance.

By combining the polymeric properties of gelatin with the chemical versatility of DES, these eutectogels can be engineered to simultaneously address the requirements of flexibility, mechanical stability, and electrochemical performance, representing a new generation of smart materials. They can be repeatedly deformed, stretched, and compressed without loss of structural integrity, while maintaining constant conductivity—essential features for soft sensors, epidermal patches, and durable bioelectrical interfaces.

In the context of previous analyses on adhesion and interfacial interactions, this chapter explores the structure, properties and applications of flexible DES–gelatin-based eutectogels, providing an integrated perspective on how these materials can combine electrical functionality, mechanical flexibility and ecological sustainability.

### 4.1. Gelatin—A Brief Overview

Gelatin is a natural biopolymer of protein origin, derived from the partial hydrolysis of collagen, which is the predominant structural protein in animal connective tissue. Due to its characteristics, gelatin has attracted considerable interest in recent decades, being a versatile material used in the food industry, as well as in the pharmaceutical, biomedical and materials science sectors. Its distinctive properties—biocompatibility, biodegradability, water solubility and the ability to form thermoreversible gels—make it a valuable natural resource for the development of new biomaterials and functional systems [86,87,88]. The raw material for gelatin comes mainly from bones, skin and connective tissue from cattle, pigs or fish. Obtaining the product may involve acid treatment (type A gelatin) or alkaline treatment (type B gelatin), with each method affecting the characteristics of the finished product [89]. Marine sources have been increasingly investigated, both for cultural and religious reasons and for their benefits associated with microbiological safety.

**Figure 6:**
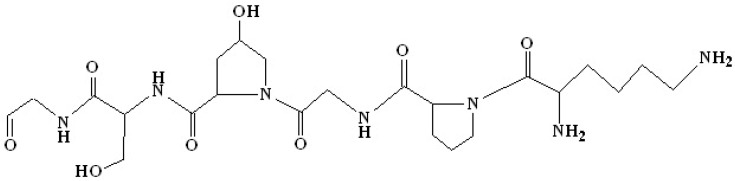
Chemical structure of a representative segment from gelatin.

The structure of gelatin is made up of polypeptide chains resulting from the partial degradation of the triple helix characteristic of collagen (Figure 6).

The unique triple helix structure of collagen is partially broken down to produce gelatin, which is extracted from the resulting mixture. Due to this process, gelatin demonstrated a wide range of functional characteristics, including the capacity to form films, the stability of emulsions and foams, gelification capabilities, and the ability to adhesion to a number of substrates [90]. The fact that gelatin contains reactive groups, which include amino, carboxyl, and hydroxyl groups, is the most important factor that contributes to its sticky characteristics. Because of their propensity to form hydrogen bonds, electrostatic interactions, or cross-linking processes, these groups have the capacity to interact with other polymers or with polar surfaces. Gelatin is a component that is advantageous in both composite systems and the manufacture of layers that stick together firmly because of these properties. These layers may play a structural or functional role [91].

Because of the physical interactions between the polypeptide chains, gelatin has the capacity to build three-dimensional networks that are hydrated, otherwise known as hydrogels. The gels produced are thermoreversible, transitioning from a sol state to a gel state based on temperature. Nevertheless, because of their limited mechanical stability and strength, hydrogels derived solely from gelatin are frequently subjected to chemical modifications or combined with other polymers. One of the most noteworthy examples is GelMA, which can produce hydrogels with improved mechanical properties and enhanced adhesion to surfaces and cells by way of photoinduced crosslinking [92]. This capacity to adhere to biological substrates is critical for applications in biomedical science because it facilitates cell attachment and extracellular matrix development. Gelatin, unlike many synthetic polymers, which are biologically inert, contains recognition sites for cells, which somewhat replicates the natural microenvironment of collagen [93].

There is a great deal of interest in gelatin hydrogels because of their gelation and adhesion capabilities. These materials are the topic of significant study for their potential uses in tissue engineering, regenerative medicine, controlled drug delivery systems, and biosensors [94]. These hydrogels are also incorporated into developing technologies, such as three-dimensional bioprinting, in which the adhesive properties of gelatin help to improve the stability of printed structures and facilitate contact with cells [95]. Because of this, gelatin is not only considered a typical gelling agent, but it is also considered a functional substance that has crucial adhesive qualities, which means that it is a key component in the creation of advanced hydrogels. This adaptability serves as confirmation that it plays a critical part in the shift from conventional natural polymers to cutting-edge biomaterials, which are capable of tackling modern-day issues in the fields of science and technology.

As a naturally occurring macromolecule, gelatin has outstanding biodegradability and biocompatibility. As a result, it is frequently employed in the manufacturing of soft products [94]. Gelatin hydrogels have been used in a variety of industries, such as 3D printing [96], drug delivery [97], and electronic devices [98]. However, the long-term uses of gelatin hydrogels are severely limited by the uncontrolled dehydration that occurs in ambient conditions. In gelatin hydrogels with elevated water content, the low polymer fraction has been shown to diminish the interaction between gelatin chains, consequently leading to reduced tensile strength and strain. Conversely, upon dehydration, there is an observed enhancement in interchain interaction, with the formation of triple helix structures of gelatin produced by crystallization playing a pivotal role in enhancing tensile strength and strain. However, it is imperative to note that excessive dehydration of the gelatin hydrogel can result in a hard and brittle material. It is therefore evident that the precise regulation of gelatin hydrogel dehydration is paramount to ensure its sustained functionality and performance.

### 4.2. Cytotoxicity and Biocompatibility of Gelatin-DES Eutectogels

Given the potential biomedical applications of gelatin–DES eutectogels, evaluation of cytotoxicity and biocompatibility is essential. Gelatin is a well-established biopolymer with widely recognized biocompatibility and biodegradability. Overall, the incorporation of DES into gelatin-based matrices may influence biological interactions by modifying ionic environments, hydrogen-bonding networks, and molecular stability within the material. DES-based eutectogels may demonstrate good cytocompatibility; nevertheless, systematic studies specifically addressing gelatin–DES hydrogels are still scarce in the current literature.

#### 4.2.1. In Vitro Cytotoxicity of DES-Based and Gelatin-Containing Hydrogels

The majority of published research regarding the cytotoxicity of DES-based hydrogels employs conventional in vitro assays such as MTT, Live/Dead staining, and cell proliferation studies. For these tests, fibroblasts, keratinocytes, and macrophages are used as model cell types. Studies show that DES formulations that include biocompatible hydrogen bond donors and acceptors, such as choline chloride, polyols, and organic acids, can keep cell viability high (>85–90%) within the right concentration ranges [48,53,99]. Injectable ionic gels made from choline chloride–ethylene glycol DES and natural polymers have been shown to be very cytocompatible. These gels have been demonstrated to enhance fibroblast and keratinocyte adhesion and proliferation without exhibiting any noticeable adverse effects [100]. Even though these systems may not directly include gelatin, they do provide a useful biological baseline for soft materials that contain DES and are meant to be used in biomedical applications. The fact that gelatin does not inherently degrade cellular compatibility is shown by the fact that gelatin-based hydrogels that do not include DES regularly demonstrate high cell viability (>80–95%) [101]. As a result, the gelatin matrix itself is not the primary factor that determines the reported cytotoxicity in gelatin–DES systems; rather, the composition, concentration, and exposure period of the DES substance are the primary determinants.

#### 4.2.2. In Vivo Biocompatibility

To date, in vivo investigations of DES-based hydrogels remain limited. Animal studies have examined wound healing and hemostatic applications utilizing DES-based ionic gels, yielding promising outcomes characterized by minimal inflammatory response, superior functional performance, and exceptional tissue compatibility [100]. There have not yet been any direct in vivo tests of hydrogels made of gelatin and DES, but these results suggest that gel systems based on DES may be able to work in biological settings.

#### 4.2.3. Effect of DES Composition on Cytocompatibility

The cytotoxicity of DES-containing systems depends on the chemical characteristics of the hydrogen bond acceptor and donor, their molar ratio, and the presence of water. DES composed of organically generated or physiologically compatible constituents (e.g., choline chloride, glycerol, lactic acid) often demonstrate reduced cytotoxicity compared to those containing more aggressive ionic species [48,53,99]. This underscores a distinct structure–toxicity connection, highlighting the need of judicious DES selection for biological applications.

#### 4.2.4. Comparative Overview of Reported Studies

This subsection provides a comparative overview of previously reported in vitro and in vivo studies on cytotoxicity and biocompatibility of DES-based and gelatin-containing hydrogel systems. Table 4 summarizes representative studies reporting cytotoxicity or biocompatibility data for gelatin-based hydrogels, DES-based gels, and related eutectogels.

In general, the research that is currently available suggests that DES-based hydrogels have the potential to demonstrate excellent cytocompatibility when they are formed with components that are physiologically tolerated. The number of systematic cytotoxicity and in vivo biocompatibility investigations that explicitly target gelatin–DES hydrogels is still very low. Standardized biological testing should be the primary focus of future research in order to completely assess the biomedical potential of these developing materials.

### 4.3. Gelatin-Based Eutectogels for the Sensitive Strain Sensor

**Figure 7:**
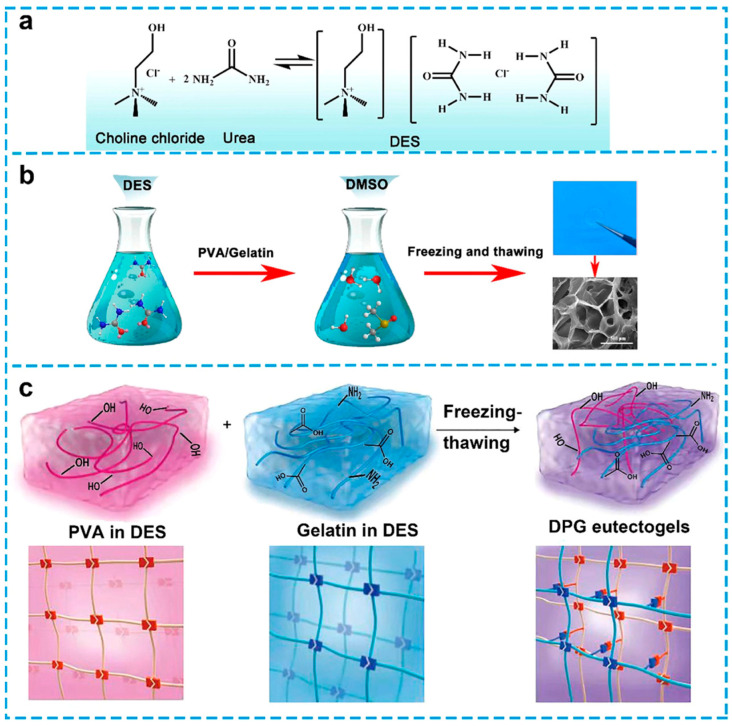
Illustrations depicting the steps involved in creating DES/PVA/gelatin (DPG) eutectogels as well as how they perform: (**a**) schematic representation for the introduction of common HBD (urea) and HBA (ChCl) in the DES production process; (**b**) schematic of the DPG eutectogels’ synthesis procedure; and (**c**) representation process by which the cross-linked network forms the DPG eutectogel. The figure is reproduced from [62].

In recent years, wearable and flexible strain sensors have garnered a lot of interest because of their potential uses in motion detection, soft robotics, human–machine interfaces, and health monitoring. The use of environmentally friendly, biocompatible, and mechanically compliant materials that can efficiently convert mechanical deformation into electrical impulses is a crucial prerequisite for these devices. In the vast array of biopolymers investigated for this purpose, gelatin has shown to be a very versatile option. To provide electrical conductivity while maintaining its elastic and flexible qualities, gelatin is frequently mixed with conductive fillers, such as graphene, metal nanoparticles, carbon nanotubes, or ionic liquids, in strain sensor design. Gelatin hydrogels’ soft and water-rich properties enable effective deformation in response to external stress, and the conductive networks included into the matrix translate these deformations into detectable changes in capacitance or resistance. It is feasible to design sensors with great sensitivity, a broad strain range, and exceptional repeatability by adjusting the gelatin content, crosslinking density, and conductive additive type. An unconventional DES system constituted by betaine and EG, was conceptualized for the fabrication of eutectogels through radical copolymerization of *N*-hydroxyethyl acrylamide (HEAA) monomer in the presence of gelatin and MXene nanosheets. To obtain the DES, betaine and EG were blended in a molar ratio of 1:3, heated, and stirred at 80 °C for approximately 2 h until clear liquids were obtained [105]. The incorporation of MXene into eutectogels has been demonstrated to enhance the mechanical properties, including tensile and compressive strength. The concentration of MXene in the eutectogels ranged from 0 to 12 mg/mL. It was observed that with an increase in the concentration of MXene, the tensile stress, elongation at break, toughness, and elastic modulus initially increased, followed by a decrease. However, experimental findings indicated that a concentration of 8 mg/mL MXene yielded the optimal tensile properties. The concern of conductive hydrogel freezing at low temperatures and water loss during prolonged storage is resolved by the addition of DES, which gives eutectogels remarkable long-term stability and anti-freezing qualities. Flexible strain, pressure, and temperature sensors made of eutectogel were created based on these properties. These multifunctional eutectogel sensors showed remarkable sensitivity throughout wide operating ranges, and they continue to function at their best even at −30 °C [105]. These results highlight the significant potential of eutectogels in a range of fields, such as electronic skin, wearable technology, personal healthcare, and human–machine interfaces. A DES gel electrolyte that is entirely supported by biopolymers has been created for possible use in ionic skin devices [103]. Simple heating and cooling procedures are followed by the addition of gelatin to the DES to create the clear, nonvolatile, and ionically conductive DES gels. In contrast to its hydrogel counterpart, the DES gel with 22% (wt.) gelatin demonstrated a remarkable stretchability, surpassing 300% strain. This is explained by the intermolecular interactions of both DES components (EG and ChCl) with the gelatin scaffold, which alter the biopolymer scaffold assembly. In addition to the aforementioned observations, a series of ionic skin prototype sensor devices were developed, utilizing DES gels that were supported by gelatin. These prototype sensors demonstrated a number of encouraging results. For instance, a pressure sensor prototype exhibited the capability to detect pressures as low as 1 kPa with high sensitivity. Furthermore, a strain sensor based on the DES gel exhibited a favorable degree of linearity in its response and a gauge factor of approximately 0.5. These characteristics enabled DES gel-containing ionic skin devices to precisely monitor multitouch inputs and finger bending on a 3 × 3 sensor array in human subjects. The results of the study [103] corroborated the viability of DES gel electrolytes supported by gelatin for use in ionic skins, and they implied that, owing to their high nonvolatility, DES gels may be a perfect alternative to ionically conductive hydrogels in a variety of applications [103]. A high-ion-conductive eutectogel with multi-environmental adaptability and recyclability was recently developed and published (Figure 7) [62].

**Figure 8:**
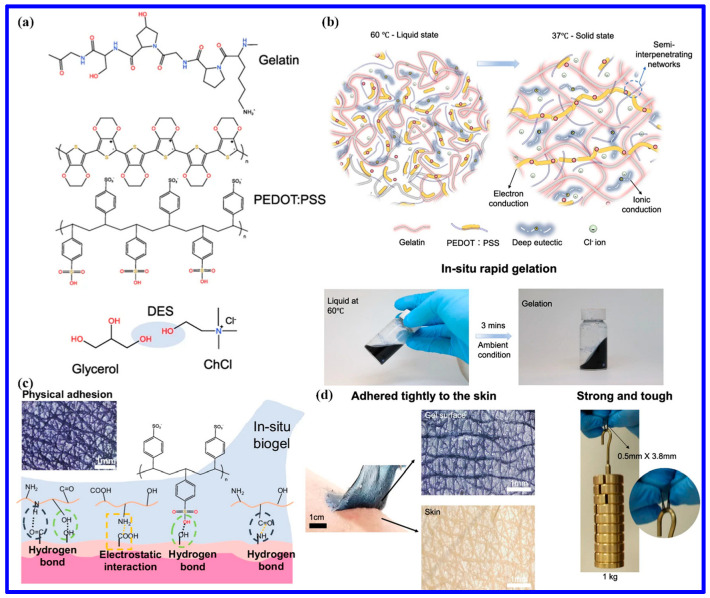
Design and main features of the in situ biogel. (**a**) The chemical constituents of the in situ biogel; (**b**) the liquid-to-solid transformation concept, accompanied by a schematic diagram and optical images that illustrate the rapid gelation process of the biogel; (**c**) The representation of the adhesion mechanism of the biogel with the skin; (**d**) The in situ biogel’s capacity to adhere to the skin along with an optical representation of a small-scale in situ biogel that shows strength and durability by supporting a sizable weight. The figure is reproduced from [106].

The material in question consists of a DES consisting of ChCl and urea, which serves as a gelator and disperser, and gelatin-strengthened poly(vinyl alcohol) (PVA). It is worthwhile to note that the eutectogel has an ultimate tensile strength of 6.8 MPa thanks to the cross-linked network created by the interaction of PVA and gelatin polymer molecules. Additionally, it has been demonstrated that the ionic conductivity of the eutectogel, measured as 0.12 S/m, is enhanced by the ionic liquid-like property of DES at ambient temperature and the number of hydrogen bonds in DES. These characteristics imply that the eutectogel has improved resistance to environmental stressors. Based on this eutectogel, a dependable strain sensor exhibits exceptional stability in hostile chemical conditions, such as bases and acids, and throughout a broad temperature range, from −20 °C to 100 °C. Furthermore, this recyclable eutectogel may be easily broken down and re-gelatted using a simple solvation–evaporation procedure, preserving its original mechanical and sensory properties. This study provides information on how to lessen the environmental effect of other electronic trash and suggests a possible use for this eutectogel in wearable electronics. The gelatin and poly(3,4-ethylenedioxythiophene (PEDOT): poly(styrenesulfonate) (PSS) solution were combined with the same DES, that included glycerol and ChCl. The authors chose DES because it has better ionic conductivity and thermal stability than water as a solvent. Through ionic and hydrogen bonding interactions, the hydroxyl groups in DES improve solubility, stability, and network formation in the polymer networks. This enhances the gel’s resilience, conductivity, and mechanical qualities. This solvent formulation also tackles the prevalent problem of water loss in conductive hydrogels, which can affect the quality and stability of signals [106]. The in situ biogel is composed of gelatin, PEDOT:PSS, and DESs, which collectively impart the qualities that define the material (Figure 8a). As demonstrated in Figure 8b, when amalgamated, the gelatin/PEDOT:PSS in DES maintains a liquid state with optimal flowability. Subsequent to application, it undergoes accelerated in situ gelation, resulting in solidification into a pliant, adhesive gel within three minutes. This characteristic renders it suitable for applications such as painting, printing, and spraying. The biogel’s noteworthy adhesive capacity is presumably attributable to its extensive hydrogen bonding and electrostatic interactions with the skin. Moreover, the biogel’s seamless compatibility with the skin contributes to the formation of a micro-texture that potentiates physical adherence (Figure 8c). The remarkable modulus (~0.3–1.1 MPa) and tensile strength (~1–3 MPa) of the semi-interpenetrating network of PEDOT:PSS and gelatin are equivalent to those of skin. Furthermore, the biogel has a dual conductive mechanism and strong adhesive qualities (~1 MPa), which produce exceptional conductivity and a high signal-to-noise ratio (SNR, ~30–40 dB) for the detection of sEMG and ECG signals (Figure 8d). The biogel is appropriate for epidermal electronics under mechanical stress because of its high strength, robust binding, and superior detecting capabilities [106].

**Figure 9:**
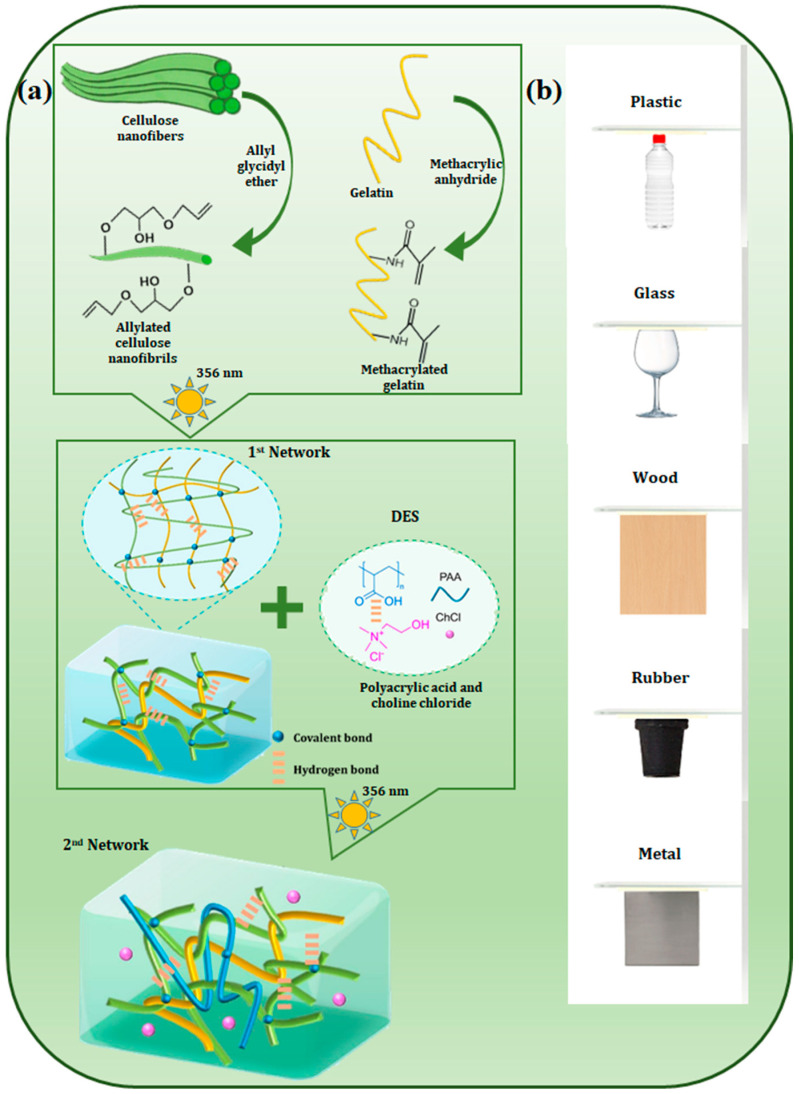
The design and structural characterization of the DN-C/G elastomer. (**a**) Diagram illustrating the synthesis process, molecular composition and intermolecular forces within the DN-C/G-Elastomer. (**b**) Digital pictures of DN-C3/G-elastomer attached on typical surfaces. The figure is adapted from [108].

A novel approach was recently reported in the literature in order to regulate the dehydration of gelatin hydrogel to a satisfactory level [107]. The authors of this study introduced hydrated ions of ChCl into the hydrogel networks. Gelatin-DES (ChCl-glycerin) based eutectogels are obtained via a solvent replacement technique that yields materials with strong anti-freezing, self-healing, and recycling properties. Additionally, the gel’s flexibility and conductivity make it suitable for use as a wearable sensor to track human movement. This method enables the precise modulation of the gelatin material’s relative water content, ranging from approximately 0.8 to 45 wt%, by adjusting the ChCl/gelatin weight ratio from 0 to 3. Notably, the retained water content is observed to persist for a minimum of 30 days under ambient conditions. However, after being dehydrated for 8 h at room temperature with a humidity of about 30%, the tensile strength of the gelatin materials drops from 20 MPa for pure gelatin to 9.5 MPa for the sample with a weight ratio of 0.5 ChCl/gelatin and finally to 0.6 MPa for the sample with a ratio of 3 ChCl/gelatin [107]. Recently, another double-network elastomer (DN-C/G-Elastomer) has been reported, Figure 9. This elastomer has excellent mechanical properties, high elasticity and fatigue-resistance. It also has robust adhesion and tolerance to a wide range of environmental conditions. This elastomer is based on a dual-network strategy involving highly entangled chains. The construction of the first crosslinked network involved the use of bio-based CNFs, which are characterized by a high density of hydroxyl groups, and gelatin, which possesses a triple helical structure [108]. A PDES, composed of ChCl/AA, was then employed as the second network. The polymer chains between the networks form chain entanglements that hydrogen bonds anchor. The ChCl quaternary ammonium groups gave it antibacterial activity, excellent interfacial adhesion (with a bonding strength to polymers of 0.7 MPa), and an ionic conductivity of 0.75 mS/cm [108]. The optimized DN-C/G-Elastomer’s outstanding overall performance makes it perfect for use in wearable strain sensors that track physiological signals (like vocal cord vibration) and human motion (like joint bending), as well as for encrypted data transmission.

The structural design of the DN-C/G-Elastomer, which is founded on several double-network connections, offers it several advantages. Excellent interfacial adhesion, environmental tolerance, and antibacterial qualities are a few of them. Because of these characteristics, it is a perfect fit for multipurpose integrated electronics intended for usage in harsh conditions. The DN-C3/G-Elastomer exhibits global adhesive qualities by adhering firmly to a variety of substrates, such as metal, glass, natural wood, and polymers (rubber, and plastics), Figure 9b. Through in situ curing, DN-C3/G-Elastomer may also create a solid adhesive layer directly onto the substrate due to its double-network layer-by-layer structure. This greatly enhances the material’s overall performance and usefulness. The lap-shear adhesive capacity of a DN-C3/G-Elastomer-bonding layer following in situ polymerization was measured using common transparent substrates, including glass, polyethylene terephthalate (PET), polymethyl methacrylate (PMMA), and polyvinyl chloride (PVC) sheets, in order to assess the in situ adhesive strength. Excellent adhesive strengths of 0.53, 0.7, 0.48, and 0.38 MPa were demonstrated by the DN-C3/G-Elastomer to glass, PET, PMMA, and PVC, respectively. These exceptional adhesive qualities are attributed to the synergy of interaction factors within the hydrogel structure: hydrogen bonds, ion-dipole interactions, electrostatic attractions and mechanical interlocking. Another double network was constructed using the same strategy as in the previous study. In this case, the hydrogel matrix is synthesized using a DES formulation composed of ChCl and acrylamide (AM) as the precursor solution, with gelatin and quaternized chitosan (QCS) serving as rigid structures. These provide abundant amino and hydroxyl functional groups that improve interfacial adhesion. This DES system improves the solubility and stability of all components and facilitates molecular diffusion, thus accelerating subsequent polymerization and cross-linking reactions. The primary cross-linked network that provides mechanical support is established during fabrication by radical polymerization of AM to form polyacrylamide (PAAm). Simultaneously, Gel and QCS are incorporated as secondary network components. The resulting eutectogel displayed enhanced mechanical properties as well as noteworthy adhesion (69.9 kPa) [109].

## 5. Conclusions

Gelatin-based eutectogels have emerged as a versatile class of soft materials that combine the physicochemical advantages of deep eutectic solvents with the biocompatibility, flexibility, and structural adaptability of gelatin. The studies reviewed demonstrate that DES plays multiple roles in these systems, acting as an ionic conductor, plasticizer, antifreeze agent, and stabilizer of the gel network, while gelatin provides the three-dimensional architecture required for mechanical integrity and interfacial adhesion. Together, these components enable materials that maintain elasticity, adhesion, and conductivity even under challenging environmental conditions. Across the literature, several structure–property relationships are evident. The balance between hydrogen bonding, ionic interactions, and dynamic sacrificial bonds is central to achieving strong and tunable adhesion. Mechanical strength and environmental stability depend strongly on the DES formulation, network density, and the presence of reinforcing agents such as PVA, CNC, or conducting polymers. These insights highlight the broad functional space that can be accessed by rationally adjusting the donor–acceptor composition of DES and the polymeric framework of the eutectogel. Despite important progress, key knowledge gaps remain. Current studies rely on heterogeneous testing protocols, which makes it difficult to compare adhesion, mechanical performance, or long-term stability across systems. The molecular mechanisms governing DES–gelatin interactions are still not fully understood, limiting predictive material design. In addition, the scalability and long-term biocompatibility of DES-based systems require further investigation. Future research should prioritize the standardization of adhesion and mechanical testing, the use of molecular modeling to clarify interaction mechanisms, and the development of DES formulations with improved biodegradability and reduced toxicity. Expanding these hybrid systems toward integrated bioelectronic platforms, wearable sensors, and environmentally sustainable devices represents a promising direction for next-generation soft materials.

## Figures and Tables

**Table 1:** Overview of representative deep eutectic solvent (DES) systems reported in the literature, including their chemical composition, molar ratios, and application domains.

DES Type/System	HBA	HBD	Molar Ratio	Main Application(s)	Reference(s)
Type I—Metal salt–based	Cl^−^ + FeCl_3_	–	1:2	High conductivity, electroplating, metallurgical processes	[18,48]
Type II—Hydrated metal salts	Cl^−^ + ZnCl_2_·× H_2_O	–	1:2	Metal extraction, catalysis	[49]
ChCl:Urea (“Reline”)	Choline chloride	Urea	1:2	Cellulose swelling, biomass pretreatment	[18,49]
ChCl:Glycerol (“Glyceline”)	Choline chloride	Glycerol	1:2	Biocompatible medium, agricultural residue pretreatment	[50]
ChCl:Ethylene glycol	Choline chloride	Ethylene glycol	1:2	Moderate delignification, improved enzymatic digestibility	[51]
ChCl:Lactic acid	Choline chloride	Lactic acid	1:1–1:15	Strong delignification, selective lignin extraction	[52]
ChCl:Oxalic acid	Choline chloride	Oxalic acid	1:2	Hemicellulose and lignin removal under mild conditions	[49]
NADES (Glucose:Lactic acid)	Glucose	Lactic acid	1:1	Extraction of natural products, biodegradable medium	[53]
NADES (Choline:Fructose)	Choline	Fructose	1:1	Extraction of thermosensitive compounds	[53]

**Table 2:** Comparative properties of eutectogels based on gelatin with adhesive and conductive properties.

Composition	Mechanical Properties	Conductivity	Adhesion	Applications	Reference
Gelatin + Poly(vinyl alcohol) + DES, combined network	Tensile strength ~6.8 MPa	~0.12 S/m (ionic)	Adhesive to skin (tested as strain sensor)	Wearable strain sensors	[62]
Gelatin (1 wt%) + DES, supramolecular interactions	Good strength despite low polymer content	Intrinsic conductivity (from DES)	Exceptional adhesion on PE/PTFE, stable at extreme temperatures	Versatile substrates, harsh environments	[63]
Gelatin + DES, one-pot preparation	Elastic, self-healing	Ionic conductive	Sufficient adhesion on skin/substrates	Electrolyte for humidity sensors	[36]
Gelatin (20% *w*/*v*) + PEDOT:LS + DES	Stable via triple helix	Mixed ionic + electronic	Lignin sulfonate enhances adhesion	3D-printable wearable sensors	[64]
Gelatin + PEDOT:PSS + genipin	Flexible	Electronic conductivity	Peel force ~0.85 N on skin	Biodegradable ECG electrodes	[65]
Gelatin + dialdehyde-TOCNF + polymethacrylate	High bonding strength (T-peel: 5.52 MPa dry, 4.71 MPa wet, wood)	Non-conductive	Strong adhesion on wood/rigid substrates	Structural adhesive	[66]
Gelatin + DES + TA@CNC	Good elasticity	Ionic	Self-adhesion tunable; adhesion energy with TA	Strain sensors; skin patches	[67]

**Table 3:** Comparative overview of gelatin–DES-based eutectogels and related DES-based hydrogel systems reported in the literature.

System Type	DES Composition	Polymer Matrix	Measurement Methods	Key Reported Properties	Reference
DES fundamental system	Choline chloride/carboxylic acids	–	Thermal analysis, spectroscopy	Low vapor pressure, extensive hydrogen bonding, suppressed evaporation	[48]
DES-based hydrogels (review)	Various DES formulations	Biopolymer-based hydrogels	DSC, rheology, thermal tests	Freezing-point depression and antifreezing behavior via donor–acceptor interactions	[82]
DES electrolytes	Various DES systems	Polymer gels	Dielectric spectroscopy, EIS	Ionic transport dominated by hydrogen-bond-assisted hopping mechanisms	[83]
DES-induced conductive hydrogels	ChCl-based DES	Polysaccharide hydrogels	DSC, conductivity tests	Combined ionic conductivity and anti-freezing stability	[85]
Supramolecular soft networks	Non-DES supramolecular systems	Soft polymer networks	Tensile tests, healing cycles	Efficient self-healing via reversible non-covalent interactions	[84]

**Table 4:** Comparative summary of reported cytotoxicity and biocompatibility studies on DES-based and gelatin-containing hydrogels.

Material System	DES/Hydrogel Composition	Biological Assay	Cell Line/Model	Key Findings	Ref.
Injectable DES-based ionic gel	ChCl:ethylene glycol + natural polymers	Cell viability, proliferation	Fibroblasts, keratinocytes	High cell viability (>90%); no cytotoxicity	[100]
DES-based hemostatic gel	DES + collagen	In vivo biocompatibility	Rat injury model	Good tissue compatibility; minimal inflammation	[100]
Gelatin-based eutectogel	Gelatin + ChCl-based DES	—	—	No biological tests reported; stable gel network	[102]
Gelatin-supported DES gel electrolyte	Gelatin (22 wt%) + DES	—	—	No cytotoxicity reported; structural stability	[103]
NADES/HPC hydrogel	NADES (organic acids) + HPC	Cell viability	Human gingival fibroblasts	Viability >85%; biocompatible	[104]
DES solution (bio-based)	ChCl + natural HBD	Cell viability	Macrophages	Viability ~97% at low concentrations	[99]
Gelatin hydrogel (reference)	Gelatin (DES-free)	MTT, Live/Dead	Fibroblasts, keratinocytes	Viability 80–95%	[101]

## Data Availability

No new data were created or analyzed in this study. Data sharing is not applicable to this article.
